# Quality assessment of systematic reviews on total hip or knee arthroplasty using mod-AMSTAR

**DOI:** 10.1186/s12874-018-0488-8

**Published:** 2018-03-16

**Authors:** Xinyu Wu, Huan Sun, Xiaoqin Zhou, Ji Wang, Jing Li

**Affiliations:** 1 Department of Evidence-based Medicine and Clinical Epidemiology, West China Hospital, Sichuan University, Chengdu, Sichuan 610041 China; 2 Department of Integrated Traditional Chinese and Western Medicine, West China Hospital, Sichuan University, Chengdu, China

**Keywords:** Total hip or knee arthroplasty, Systematic review, Bibliographical characteristics, Methodological quality

## Abstract

**Background:**

Increasing numbers of systematic reviews (SRs) on total knee arthroplasty (TKA) and total hip arthroplasty (THA) have been published in recent years, but their quality has been unclear. The purpose of this study is to evaluate the methodological quality of SRs on TKA and THA.

**Methods:**

We searched Ovid-Medline, Ovid-Embase, Cochrane Databases (including HTA, DARE, and CDSR), CBM, CNKI, Wang Fang, and VIP, from January 2014 to December 2015 for THA and TKA. The quality of SRs was assessed using the modified 25-item “Assessment of Multiple Systematic Reviews” (mod-AMSTAR) tool, which was based on the AMSTAR scale. A T-test, nonparametric test, and linear regression were conducted to assess the relationship between bibliographical characteristics and methodological quality.

**Results:**

Sixty-three SRs were included, from which the majority of SRs (50, 79.4%) were conducted in Asia. Only 4 reviews were rated as high quality, and most were weak in providing a priori design (6, 9.5%), not limiting the publication type (8, 13%), providing an excluded primary studies list (4, 6.3%) and reporting support for the included primary studies (1, 1.6%). Reviews published in English journals performed better than did Chinese journals in duplicate data extraction (81.3% vs 46.7%, *p* = 0.017; 70.8% vs 33.3%, *p* = 0.009) and providing source of support for the SR (87.5% vs 33.3%, *P* < 0.001). Reviews published in journals with a higher impact factor were associated with a higher mod-AMSTAR score (regression coefficient: 0.38, 95%CI: 0.11–0.65; *P* = 0.006).

**Conclusion:**

The methodological quality of the included SRs is far from satisfactory. Authors of SRs should conform to the recommendations outlined in the mod-AMSTAR items. Areas needing improvement were providing a priori design, not limiting the publication type, providing an excluded primary studies list, and reporting conflicts of interest.

**Electronic supplementary material:**

The online version of this article (10.1186/s12874-018-0488-8) contains supplementary material, which is available to authorized users.

## Background

Keeping up with information in health care is difficult because at least 75 trials are published every day [1]. Systematic reviews (SRs) involve the synthesis of the best current evidence to address clinical questions [2] and are considered a convenient way to follow the frontier of medical practice [3]. However, they have been found to be of varying quality [4–8], which can lead to confusion [9, 10]. The quality of SRs involves their methodological quality (how well a study has been conducted) and reporting quality (how well the reviewers have reported their methodology and findings). Methodological quality is defined as the extent to which the design of an SR is capable of generating unbiased results [11]. Flaws in methodological quality may lead to bias or uncertainty about the authenticity of the results of the SR, which may mislead clinical practice and decision-making. Thus, users of SRs must be critical and prudent about the quality of the available reviews [9].

As the population continues to age [12], osteoarthritis (OA), as one of the ten most disabling diseases in developed countries, is gaining increased attention [13]. Joint arthroplasty, including total hip arthroplasty (THA) and total knee joint arthroplasty (TKA), is the ultimate treatment for osteoarthritis [14]. From 2005 to 2015, the number of randomized controlled trials of TKA and THA nearly doubled, and the number of meta-analyses increased nearly 9.5 times, from 15 in 2005 to 142 in 2015 [15, 16]. Although there have been numerous SRs on THA/TKA, it has been unclear whether the quality of the reviews was satisfactory. Therefore, the purpose of this study is to assess the methodological quality of SRs in THA/TKA and to examine the relationship between bibliographical characteristics and the methodological quality of reviews.

## Methods

Prior to beginning the review, a protocol was produced outlining the search strategy, inclusion criteria, and outcomes of interest. The protocol and changes in the review compared with the protocol are in Additional file 1: Appendix 1. Detailed information on the methodology is as follows.

### Inclusion and exclusion criteria

SRs are defined as a type of literature review that critically appraises and formally synthesizes the best existing evidence to provide a statement of conclusion to resolve specific clinical problems. Moreover, a meta-analysis involves the use of statistical methods to summarize the results of independent studies and can provide more precise estimates of health care than those derived from individual studies included within a review [2]. All studies where the authors claimed to be conducting SRs or meta-analyses and focused on the effects and safety of procedures and prostheses in primary THA or TKA, published in English or Chinese, from 2014 to 2015, were included. There were no limitations on the type of clinical settings or study populations.

### Search strategy

A search of Ovid-Medline, Ovid-Embase, Cochrane Database of Systematic Review (CDSR), Health Technology Assessment Database (HTA), Database of Abstracts of Reviews of Effects (DARE), and Chinese databases (Chinese Biomedical Literature Database (CBM), China National Knowledge Infrastructure (CNKI), Wan Fang Data, and VIP database) was conducted from January 2014 to December 2015. The reference lists of all identified relevant reviews were searched. The full search strategies can be found in Additional file 2: Appendix 2.

### Study selection and data extraction

Two reviewers (XW, HS) independently scanned the title and abstract of the studies to select eligible SRs based on the inclusion and exclusion criteria and extracted the data using a prior designed form. Any disagreement in the process of study selection or data collection was discussed, resolved by consensus, or determined with a third reviewer (JL). Ten bibliographical characteristics that have been suggested to influence the methodological quality of SRs from previous studies [6, 17, 18] and mod-AMSTAR sub-items were collected for each eligible review. We retrieved the impact factors (IFs) of the included reviews by searching the Journal Citation Reports in Web of Science (reviews published in English) and CNKI (reviews published in Chinese), specifically the IFs of the corresponding review publication year. Detailed information on mod-AMSTAR and the pre-designed bibliographical characteristics questionnaire are displayed in Table 1 and Additional file 3: Appendix 3.

**Table 1:** Methodological quality

AMSTAR Checklist	modified AMSTAR Checklist	“YES” N (%)	“NO” N (%)	“Cannot answer” N (%)
1. Was an ‘a priori’ design provided?		6 (9.5)	0	57 (90.5)
2. Was there duplicate study selection and data extraction?	2.1 Were there at least two independent data extractors for study selection?	45 (71.4)	4 (6.3)	14 (22.2)
	2.2 Was there a consensus procedure for disagreements in study selection?	38 (60.3)	5 (7.9)	20 (31.7)
	2.3 Were there at least two independent data extractors for data extraction?	46 (73.0)	3 (4.8)	14 (22.2)
	2.4 Was there a consensus procedure for disagreements in data extraction?	39 (61.9)	5 (7.9)	20 (31.7)
3. Was a comprehensive literature search performed?	3.1 Were there at least 2 electronic sources searched?	62 (98.4)	1 (1.6)	0
	3.2 Did the report include search years?	61 (96.8)	1 (1.6)	1 (1.6)
	3.3 Were key words and/or MESH terms stated and where feasible the search strategy provided?	61 (96.8)	2 (3.2)	0
	3.4 Were there supplementary searches?	49 (77.8)	9 (14.2)	5 (7.9)
4. Was the status of publication (i.e., gray literature) used as an inclusion criterion?	4.1 Were there any restrictions for publication type?	8 (13.0)	36 (57.1)	19 (29.7)
	4.2 Were there any restrictions for language?	22 (34.4)	25 (39.1)	17 (30.2)
5. Was a list of studies provided?	5.1 Was a list of included studies provided?	63 (100)	0	0
	5.2 Was a list of excluded studies provided?	4 (6.3)	59 (93.7)	0
6. Were the characteristics of the included studies provided?	6.1 Were the demographics of the participants provided?	52 (82.5)	11 (17.4)	0
	6.2 Were the characteristics of the interventions provided?	59 (93.7)	4 (6.4)	0
	6.3 Were the characteristics of the outcomes provided?	40 (63.5)	23 (36.5)	0
7. Was the scientific quality of the included studies assessed and documented?	7.1 Were there ‘a priori’ methods of assessment being provided?	55 (87.3)	8 (12.7)	0
	7.2 Was a “risk of bias” table shown in a graphic form?	55 (87.3)	8 (12.7)	0
8. Was the scientific quality of the included studies used appropriately in formulating conclusions?	8.1 Were the results of the methodological rigor and scientific quality considered in the analysis of the review?	35 (55.6)	26 (41.7)	2 (3.2)
	8.2 Were the results of the methodological rigor and scientific quality considered in the conclusions of the review?	37 (58.7)	22 (34.9)	4 (6.3)
9. Were the methods used to combine the findings of studies appropriate?	9.1 Was the homogeneity test (i.e., Chi-squared test for homogeneity, I^2^) conducted when pooling results?	61 (96.8)	2 (3.2)	0
	9.2 Was a random effects model used and/or the clinical appropriateness of combing taken into consideration when heterogeneity exists?	61 (96.8)	2 (3.2)	0
10. Was the likelihood of publication bias assessed?		20 (31.7)	42 (66.7)	1 (1.6)
11. Was the conflict of interest stated?	11.1 Were the sources of support for the SR reported?	47 (74.6)	16 (25.4)	0
	11.2 Were the sources of support for the included primary studies reported?	1 (1.6)	62 (98.4)	0

### Quality assessment

Methodological quality was assessed using the modified AMSTAR (mod-AMSTAR), which was based on the AMSTAR scale. AMSTAR is a freely accessible, validated tool for assessing the methodological quality of SRs [19]. Because some AMSTAR items contain several aspects, we refined the 11 items into 25 sub-items (Table 2). In the original AMSTAR scale, the total score was calculated by summing one point for each “yes” and zero points for “no” or “can’t answer”, resulting in summary scores ranging from 0 to 11 [20]. In our study, the total score remained the same as in the original AMSTAR because we divided the score of each item into all its sub-items. The methodological quality of the reviews was graded as high (8–11), medium (4–7) or low (0–3) quality. Our modified AMSTAR referenced the methods of Pollock and Kung [21, 22], but the modifications we made differed from theirs.

**Table 2:** Comparison between SRs on total hip/knee arthroplasty in Chinese and English journal

AMSTAR item	Total reviews marched the item (*n* = 63)	Reviews marched the item in Chinese journal (*n* = 15)	Reviews marched the item in English journal (*n* = 48)	*P* value
1	6 (9.5%)	0	6 (12.5)	0.181△
2.1	45 (71.4%)	12 (80%)	32 (66.7%)	0.520△
2.2	38 (60.3%)	11 (73.3%)	26 (54.2%)	0.188
2.3	46 (73.0%)	7 (46.7%)	39 (81.3%)	0.017^*^△
2.4	39 (61.9%)	5 (33.3%)	34 (70.8%)	0.009^*^
3.1	62 (98.4%)	15 (100%)	47 (97.9%)	1.000△
3.2	61 (96.8%)	15 (100%)	46 (95.8%)	1.000△
3.3	61 (96.8%)	15 (100%)	46 (95.8%)	1.000△
3.4	50 (79.4%)	12 (80%)	38 (79.2%)	1.000△
4.1	9 (14.3%)	0	8 (16.7%)	0.181△
4.2	22 (34.9%)	3 (20%)	19 (39.6%)	0.165
5.1	63 (100%)	15 (100%)	48 (100%)	–
5.2	4 (6.3%)	0	4 (8.3%)	0.564△
6.1	52 (82.5%)	13 (86.7%)	39 (81.3%)	1.000△
6.2	59 (93.7%)	13 (86.7%)	46 (95.8%)	0.238△
6.3	40 (63.5%)	9 (60%)	31 (64.6%)	0.748
7.1	56 (88.9%)	14 (93.3%)	42 (87.5%)	1.000△
7.2	55 (87.3%)	13 (86.7%)	42 (87.5%)	1.000△
8.1	35 (55.6%)	8 (53.3%)	27 (56.3%)	0.843
8.2	37 (58.7%)	12 (80%)	25 (52.1%)	0.055
9.1	61 (96.8%)	15 (100%)	46 (95.8%)	1.000△
9.2	61 (96.8%)	15 (100%)	46 (95.8%)	1.000△
10	21 (33.3%)	5 (33.3%)	16 (33.3%)	1.000
11.1	47 (74.6%)	5 (33.3%)	42 (87.5)	0.000^*^△
11.2	1 (1.6%)	0	1 (2.1%)	1.000△

△non-parametric test, ^*^statistically significant

The quality assessment was conducted by two of our reviewers (XW, HS). The Cohen kappa (κ) statistic was used to test for inter-observer agreement. Values of 0.01–0.20, 0.21–0.40, 0.41–0.60, 0.61–0.80, and 0.81–0.90 were considered slight, fair, moderate, substantial, and almost perfect agreement, respectively [23].

### Statistical analysis

Data were summarized as frequencies or percentages for categorical variables and as mean ± standard deviation or median (interquartile range: the 25th to 75th percentile) for continuous mod-AMSTAR score. T-tests and non-parametric tests were used to compare the quality score of SRs published in Chinese and English and to test the association between bibliographical characteristics and the total score of mod-AMSTAR. The association among the number of authors, the number of databases searched, the impact factor of the published journals and mod-AMSTAR score for each study was analyzed by a linear regression test. Scatterplot and linear regression equations were displayed for statistically significant variables. Regression coefficients (rounded to two decimal points) and 95% confidence intervals of the linear regression equation were calculated. Statistical analysis was conducted using IBM SPSS 21.0, with a two-tailed significance level of 0.05.

## Results

### Search results

**Fig. 1:**
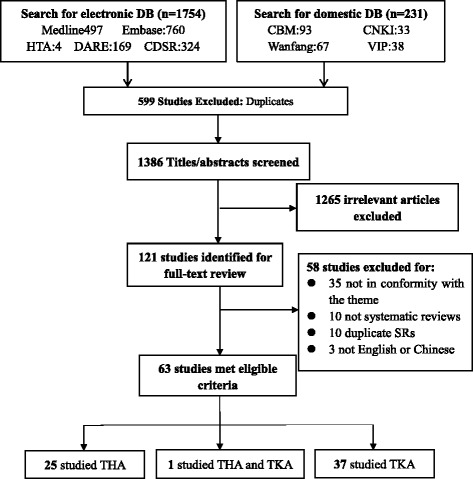
Study flowchart, which was referred to the PRISMA statement [24] (Study flow chart)

A PRISMA-like flow was utilized to demonstrate the study selection process (Fig. 1) [24]. The search strategy identified 1985 records, including 1754 from English databases and 231 from Chinese databases. After excluding 599 duplicates, screening of titles and abstracts led to the further exclusion of 1265 records. Of the 121 full-text articles retrieved, 58 were excluded, and 63 were eligible for data extraction. Inter-rater agreement between two assessors for the mod-AMSTAR assessment was almost perfect (κ = 0.895, *p* < 0.001). Detailed information of the included articles is displayed in Additional file 4: Appendix 4.

### Methodological quality

In general, the included studies were more likely to have searched two or more databases (Item 3), provided a list of the included primary studies (Item 5.1), provided the characteristics of the participants and interventions (Item 6.1 and Item 6.2), assessed and documented the scientific quality of the included studies (Item 7) and provided appropriate methods to combine the findings (Item 9), but they were less likely to have provided an a priori design or a published protocol (Item 1), not limited the publication type (Item 4.1), provided an excluded primary studies list (Item 5.2) and reported support for the included primary studies (Item 11.2) (Table 1). The overall mean score for all 63 included reviews was 6.336 ± 1.225 (range from 3 to 10), and the median mod-AMSTAR score was 6.17 (IQR 5.5–7.46). Specifically, 4 reviews were rated as high quality [25–28], 58 as moderate quality, and 1 as low quality [29]. A list of the included SRs and detailed mod-AMSTAR assessments are shown in Additional file 3: Appendix 3.

### Comparison between Chinese journals and English journals

There were 15 articles (23.8%) published in Chinese journals and 48 (76.2%) published in English journals. The methodological quality of reviews published in English journals was better than that of reviews in Chinese journals, especially in duplicating data extraction and providing sources of support for the SR (Table 2).

### Bibliographical characteristics and methodological quality

We described and tested 10 bibliographical characteristics that could have influenced the methodological quality of the reviews. The proportions of reviews published in 2014 (47.6%) and 2015 (52.4%) were almost equal. The quantity of reviews on TKA (37, 58.7%) was more than that of THA (25, 39.7%). Over half of the reviews were conducted by teams based in Asia (79.4%). The reviews searched a median of 4.5 databases, and only 20.6% searched non-English databases. All SRs included randomized controlled trials (RCTs), and 41.3% included observational studies. Details about the bibliographical characteristics of the included reviews are shown in Table 3.

**Table 3:** Association between publication characteristics and methodological quality of SRs on total hip/knee arthroplasty

Bibliographical characteristics	Number (%)	AMSTAR score	Results
1、published year			
2014	30 (47.6%)	6.40 ± 1.35	*P* = 0.375
2015	33 (52.4%)	6.32 ± 1.19	
2、surgical type			
THA	25 (39.7%)	6.08 (5.46–7.09)^b^	△*P* = 0.435
TKA	37 (58.7%)	6.42 (5.58–7.50)^b^	
THA and TKA	1 (1.59%)	–	
3、language			
Chinese	15 (23.8%)	6.67 (5.17–6.08)^b^	△*P* = 0.052
English	48 (76.2%)	5.67 (5.67–7.50)^b^	
4、location of the corresponding author			
Asia	50 (79.4%)	6.17 (5.5–7.19)^b^	△*P* = 0.098
America	9 (14.3%)	6.09 (4.34–7.84)^b^	
Europe	4 (6.3%)	7.75 (6.37–9.5)^b^	
5、Number of SRs that included a PRISMA-like flow			
Included a PRISMA-like flow	53 (84.1%)	6.48 ± 1.20	*P* = 0.752
Did not include a PRISMA-like flow	10 (15.9%)	5.73 ± 1.45	
6、Was the SRs published in journal			
Number of SRs published in journal	59 (93.7%)	6.17 (5.50–7.50)^b^	△*P* = 0.903
Number of SRs not published in journal	4 (6.3%)	6.34 (5.62–7.29)^b^	
7、Tools for assessing risk of bias of primary studies			
Cochrane	35 (55.6%)	6.67 (5.50–7.50)^b^	△*P* = 0.312
Jadad scale	11 (17.5%)	5.75 (5.67–6.75)^b^	
The PEDro scale	2 (3.2%)	7.46	
Other single assessment tool	7 (11.1%)	6.00 (4.5–7.00)^b^	
Two or more assessment tools	6 (9.5%)	7.00 (5.63–8.13)^b^	
Not reported	2 (3.2%)	5.45	
	Average(range)		
8、Total number of authors in SRs	4.9 (1–8)	–	#Nonlinear relation
9、Number of databases searched	4.5 (1–12)	–	#Nonlinear relation
10、Median impact factor of the journal for which the included study was published	1.864 (0.293–5.228)	–	#P = 0.006^*^

Values in AMSTAR score are mean ± standard deviation except for ^b^median (the 25th and 75th percentile). Values in results are tested in t-test except for: △non-parametric test; #linear regression test. ^*^statistically significant

**Fig. 2:**
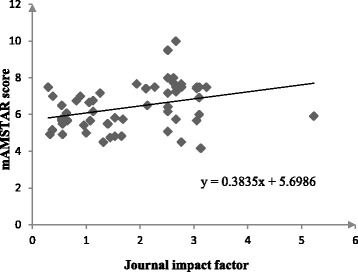
Relationship between mod-AMSTAR score and journal impact factor (Relationship)

Our analysis demonstrated that reviews published in higher impact factor journals were significantly associated with a higher methodological quality (regression coefficient: 0.38, 95%CI: 0.11–0.65; *P* = 0.006). The linear regression trend is shown in Fig. 2.

## Discussion

### Literature search

Although the same search words were used for both English and Chinese databases, the corresponding search strategy seemed to be more sensitive in searching English databases than in Chinese databases, with 7.6 times more studies found in English than were found in Chinese. Even though the quantity of studies ineligible for inclusion from English databases (1754) was higher than that from Chinese (216), it resulted in 3 times more English studies than Chinese studies being eligible for our study.

### Overall methodological quality assessment

Our study assessed the methodological quality of 63 SRs on total hip and knee arthroplasty published from 2014 to 2015. The overall methodological quality of SRs on THA and TKA is better than that of other medical fields such as nursing, oral health, hand and wrist pathology [6, 30, 31], but the proportion of reviews with high methodological quality (6.3%) is less than that of those fields. Only four reviews were of high quality, whereas most were of moderate quality (58, 92.1%). Few reviews adequately satisfied the quality items, such as the use of a priori design, not limiting the publication type, providing a list of excluded primary studies, and reporting the sources of financial support for the included primary studies. Users of SRs on THA or TKA should be more cautious, and reviewers should focus more on improving the quality instead of quantities of SRs.

In our study, only six reviews were identified to have a priori design (9.5%) [25–28, 32, 33], of which three had registered or published their a priori designs (4.8%) [26–28]. Reviews on oral health, urology, and hand and wrist pathology also performed poorly in this item [30, 31, 34]. When The Cochrane Collaboration was set up in 1993, it required authors to register a review proposal form before conducting SRs to avoid publication bias and duplicate research [35]. Non-Cochrane reviews should have their a priori design registered in a formal registry platform such as PROSPERO (international prospective register of systematic reviews) [36], as PRISMA (Preferred Reporting Items for Systematic Reviews and Meta-Analyses) has suggested [24], or should publish their protocol in appropriate journals.

Only 8 (13%) eligible reviews did not limit the study publication type [27, 28, 37–42], which was similar to the fields of nursing, urology, hand and wrist pathology [6, 31, 34]. In most cases, studies containing significant findings were more likely to be published than were those with non-significant findings, and SRs based mainly on the published literature tended to overestimate the efficacy of interventions [43–45]. Restricting the study publication type may leave out unpublished literature and/or gray literature and may cause publication and query bias. Treatment effects can be overestimated in cases of publication bias, even when the included individual trials have a low risk of bias [33]. Therefore, all types of publications should be included to avoid confusion.

Only four included studies provided their list of excluded studies (6.3%) [41, 46–48], which was inferior to most other medical fields, except for nursing, pulmonary and diabetes mellitus treatment [5, 6, 18]. Journals generally limit the space available to publish the list of excluded studies, but some provide unlimited space (often online) to publish the list of excluded studies as supplementary material.

Another area of concern is the lack of reporting surrounding conflicts of interest (COIs). While one review reported funding sources for all the included primary studies [49], this was not the case in reviews of other fields, such as pulmonary, hand and wrist pathology, urology, diabetes mellitus treatment and burn care [5, 10, 18, 31, 50]. Previous studies have clearly shown the relationship between industry funding and positive results from meta-analyses [51, 52]. COIs related to the funding of biomedical research by pharmaceutical companies and the financial relationships between researchers and pharmaceutical companies may influence the framing of research questions, study design, data analysis, interpretation of findings, whether to publish the results and what results are reported. Compared with non-industry-funded trials, pharmaceutical industry-funded studies more often yield results or conclusions that support the sponsor’s drug [53, 54], so detailed information on COI should be reported. For an impartial assessment, researchers could list the funding sources of the included studies in table form.

### Methodological quality assessment between SRs in Chinese and English

The methodological quality of reviews published in English is better than that of Chinese in duplicate data extraction and reporting sources of support for the SR. To improve the quality of SRs in Chinese, we suggest that Chinese authors who plan to conduct SRs be formally trained on the methodology of SRs and that editors of Chinese journals should adopt AMSTAR in reviewing the manuscripts of SRs.

### Quality assessment scale of primary studies

SRs or meta-analyses of invalid studies may produce misleading results. Evaluating the validity of the included studies is therefore an essential component of a review. The proper tools should be used to assess the risk of bias of the included studies in a review. The Cochrane Collaboration’s tool for risk of bias (55.6%) and the Jadad Scale (17.5%) are the most commonly adopted tools for assessing the risk of bias of RCTs in our study. However, the use of the Jadad scales for assessing the quality or risk of bias has been explicitly discouraged in Cochrane reviews because it places a strong emphasis on reporting rather than conducting quality and does not cover one of the most important potential biases in randomized trials: allocation concealment. The Cochrane Collaboration recommends a specific tool for assessing the risk of bias in RCTs that addresses seven specific domains: sequence generation, allocation concealment, blinding of participants and personnel, blinding of outcome assessment, incomplete outcome data, selective outcome reporting and ‘other issues’ that do not fit into these categories.

Although there was no consensus, most reviews assessed the quality of the included primary observational studies, such as cohort and case-control studies, using the Newcastle-Ottawa Scale (NOS). However, the inter-rater reliability [55] and validity [56, 57] of this scale have been questioned. Further, it has been argued that quality summary scores may mask variations in quality by domain and use an unclear, often implicit, weighting scheme [58, 59]. A tool for Risk Of Bias in Non-randomized Studies of Interventions (ROBINS-I) was developed for evaluating the risk of bias in estimates of the comparative effectiveness (harm or benefit) of interventions from studies that did not use randomization to allocate units (individuals or clusters of individuals) to comparison groups, including observational studies such as cohort studies, case-control studies, and quasi-randomized studies. The tool is particularly useful for those undertaking SRs that include non-randomized studies [60].

### Association between publication characteristics and methodological quality

We found that among the collected bibliographical characteristics, the impact factors of the published journals can affect the methodological quality of reviews. Linear regression analysis showed that having a higher impact factor is associated with a higher mod-AMSTAR score; this finding is similar to a previous study by Fleming [61]. It is likely that reviews with better methodological quality are more readily accepted by higher impact factor journals.

### Strength and limitations

The present study is the first to comprehensively assess the methodological quality of SRs on total hip or knee arthroplasty. Moreover, the AMSTAR scale was refined, which allowed the methodological flaws of the included reviews to be more accurately identified. The recently published AMSTAR 2 (an update of AMSTAR) supports this refining [62]. AMSTAR 2 not only provides a “partial Yes” response in some instances where it was considered worthwhile to identify partial adherence to the standard but also splits some items that contain more than one idea, such as splitting items 2 and 5 in the original AMSTAR into items 5 and 6, 7 and 8, respectively, in AMSTAR 2.

This study has some limitations. First, it only included reviews published in English and Chinese, so bias could be introduced if well-conducted reviews are more likely to be reported in an international, English journal whereas less well-conducted reviews are published in a local journal, and studies published in these two languages may differ from studies in other languages. Second, it did not assess the reporting quality of the included reviews. The AMSTAR appraisal process is difficult to implement when the reporting quality is poor. Items that are judged as “Cannot answer” may contain important information that the authors do not describe in detail (Table 1). This can be attributed to space restrictions in print journals. Authors are encouraged to adhere to the PRISMA requirement to report all important components of SRs. Third, it merely included studies published in 2014 or 2015 due to lack of resources. This can present a bias, as the quality of more recent studies is likely higher than that of older studies. Fourth, although AMSTAR is a reliable and valid tool for assessing the methodological quality of SRs, the AMSTAR score has not been validated in any studies [63, 64]. The study modified AMSTAR but did not validate it. In addition, the mod-AMSTAR score generally exceeds the AMSTAR score; some items could receive a partial score with mod-AMSTAR (e.g., 0.25, 0.67) but a score of 0 on AMSTAR if they did not meet all the criteria required to obtain a point. This could lead to substantial differences between AMSTAR and mod-AMSTAR scores, with more reviews judged as having higher quality by mod-AMSTAR than by AMSTAR, resulting in bias when the results are compared with those of other studies. Moreover, the practical inclusion criteria for SRs could miss relevant SRs that were not clearly stated or included reviews that are not SRs. Future studies should cover the relevant reviews based on a clear SR definition.

## Conclusion

The study demonstrates that the methodological quality of SRs on total TKA and THA is far from satisfactory. Areas that require improvement in the future include providing a priori design, not limiting the publication type, providing an excluded primary studies list, and reporting COIs. However, the AMSTAR score can only reflect the methodological quality of the SR, namely, the internal validity. Therefore, a review with a higher AMSTAR score would have more valid results. However, the extent to which a review is capable of affecting practice depends on the clinical importance of the results and the generalizability of the review. Clinicians should be judicious when applying the conclusions of the SRs results to their own patients. Authors, journal editors and peer reviewers have an important role in ensuring the continuous improvement of SR quality by adopting the methodological and reporting standards of AMSTAR and PRISMA.

## Additional files

Additional file 1: **Appendix 1**. Protocol: the protocol of this study. (Protocol). (DOCX 27 kb)

Additional file 2: **Appendix 2**. Search strategies: Detailed information on search strategies of this study in Medline, Embase, Cochrane Databases (including HTA, DARE and CDSR), CBM, CNKI, Wang Fang and VIP. (Search strategies). (DOCX 19 kb)

Additional file 3: **Appendix 3**. AMSTAR score and list of included reviews: mod-AMSTAR score for each study and reference information of all included studies. (AMSTAR score and list of included reviews). (DOCX 58 kb)

Additional file 4: **Appendix 4**. Data extraction table: Extraction items and results of each study. (Data extraction table). (XLSX 21 kb)

## Abbreviations

AMSTAR: Assessing the Methodological Quality of Systematic Reviews
IQR: Interquartile range
mod-AMSTAR: Modified AMSTAR
RCT: Randomized control trial
SR: Systematic review
THA: Total hip arthroplasty
TKA: Total knee arthroplasty
