# Supplementary material for: Quality assessment of systematic reviews on total hip or knee arthroplasty using mod-AMSTAR
Source: BMC Med Res Methodol. 2018 Mar 16;18:30. doi: 10.1186/s12874-018-0488-8 (PMC5857117; doi:10.1186/s12874-018-0488-8)
Supplement: Supplementary file 1 — Appendix 1. Protocol: the protocol of this study. (Protocol). (DOCX 27 kb) [file 12874_2018_488_MOESM1_ESM.docx]

Quality assessment of systematic reviews of total hip or knee arthroplasty using AMSTAR: the Protocol

**Authors:** Xinyu Wu, Huan Sun, Ji Wang, Jing Li

**Contact person:** Jing Li; [lijing68@hotmail.com](mailto:lijing68@hotmail.com)

**Affiliation:**

1. Chinese Cochrane Centre, West China Hospital, Sichuan University, Chengdu, Sichuan, China**;**
2. Department of Integrated Traditional Chinese and Western Medicine, West China Hospital, Sichuan University Chengdu, China.

**Anticipated or actual start date: 2017/03/01**

**Anticipated completion date: 2017/06/20**

**Backgrounds**

Total joint arthroplasty(TJA) is the ultimate treatment of osteoarthritis, while total hip arthroplasty (THA) and total knee joint arthroplasty (TKA) are most commonly used procedures within TJA. Currently, studies (including systematic reviews) on THA and TKA are on the rise, yet the quality of those studies is not clear. Systematic review is regarded as the highest level of evidence for clinical practice, but it was also found to be varying quality, which may mislead clinical practice and decision-making.

**Objectives**

1. Using AMSTAR (Assessment of Multiple Systematic Reviews) Scale to assess the methodological quality of systematic reviews in total hip or knee arthroplasty
2. To examine the relationship of bibliographical characteristics with the methodological quality of reviews.

**Methods:**

1. **Eligible criteria for studies:**

systematic reviews or meta-analyses which focuses on the effects and safety of procedures and prostheses in primary THA or TKA published in English or Chinese from 2014 to 2015. There are no limitation on the type of clinical setting and study population.

1. **Search methods for identification of studies:**

A comprehensive searched will be conducted on Ovid-Medline, Ovid-Embase, Cochrane Databases (including HTA, DARE and CDSR), CBM, CNKI, Wangfang and VIP from January 2014 to December 2015. We will use search words "Hip arthroplasty", "knee arthroplasty", "hip replacement", "knee replacement", "hip joint", "knee joint", "Prostheses", "Joint Prosthesis", "Prostheses" or "Implants" to find studies related to the procedures and prostheses in THA or TKA and combine words "systematic review" or "meta-analysis" to search systematic reviews.

1. **Study selection and data extraction:**

Results of database searches will be imported into Endnote X7 reference-handling system, two reviewers (XW and HS) will independently use this tool to select the studies by scanning study titles and, if available, abstracts, to determine the potential eligible studies. After obtaining full reports of candidate studies, the same reviewers will check the eligibility of each study through full text review. Data extraction will be conducted by the same reviewers, they will independently collect information from each eligible study. Any disagreement in study selection or data collection will be discussed and resolved by consensus, or with a third reviewer (JL).

The primary outcomes we will collect are 15 bibliographical characteristics claimed to influence the methodological quality of SR from previous studies and 25 modified AMSTAR items. All the data will be collected in the Microsoft Excel 2010. The 15 characteristics are as follows:

1. published year: 2014; 2015
2. surgical type: TKA; THA; TKA and THA
3. language: Chinese; English
4. location of corresponding author: Asia; America; Europe
5. number of SRs that searched specific databases:

Searched international databases; searched non-English databases

1. Search terms reported for one or more electronic databases:

Topics/free text/keywords/MeSH; Full Boolean

1. Eligibility of study design:

RCT only or RCT and observational study

1. language of restriction:

Included English publication only; English and language other than English; Language criteria not reported

1. Number of SRs that included a PRISMA-like flow:

Included a PRISMA-like flow; do not included a PRISMA-like flow

1. Conflicts of interest:

Number of SRs that declared conflicts of interest; Number of SRs that declared no conflicts of interest; Number of SRs that did not mention conflicts of interest

1. Do the SRs published in journal:

Number of SRs published in journal; Number of SRs not published in journal

1. Tools for assessing risk of bias of primary studies:

Cochrane; Jadad scale; The PEDro scale; Other single assessment tool; Two or more assessment tools; Not reported

1. Total number of participants in SRs
2. Number of databases searched
3. Median impact factor of the journal for which the included study was published
4. **Study quality assessment**

Methodological quality will be assessed by the modified AMSTAR (mASTAR). AMSTAR is a freely accessible validated tool for assessing the methodological quality of SR, but some of the items contain several aspects, to better detect the methodological flaw in included studies, we refined the 11 items AMSTAR scale into 25 sub-items. Score for the original AMSTAR scale will be calculated by summing one point for each “yes” and no point for “no”, “can’t answer”, resulting in summary scores from 0 to 11. Our study will not change the total score by equally dividing the score of each item to all its sub-items.

The Cohen kappa (κ) statistic will be used to test for inter-observer agreement. Value of 0.01 to 0.20, 0.21 to 0.40, 0.41 to 0.60, 0.61 to 0.80, 0.81 to 0.90 was considered slight, fair, moderate, substantial, and almost perfect agreement, respectively.

The detailed information of modified ASMTAR scale is displayed as follows:

**Modified AMSTAR Checklist:**

| **modified AMSTAR Checklist** | **YES** | **NO** | **Cannot answer** |
| --- | --- | --- | --- |
| 1. Was an 'a priori' design provided? |  |  |  |
| 2.1 Was there at least two independent data extractor for study selection？ |  |  |  |
| 2.2 Was there a consensus procedure for disagreements in study selection？ |  |  |  |
| 2.3 Was there at least two independent data extractor for data extraction？ |  |  |  |
| 2.4 Was there a consensus procedure for disagreements in data extraction？ |  |  |  |
| 3.1 Was there at least 2 electronic sources searched？ |  |  |  |
| 3.2 Did the report include search years? |  |  |  |
| 3.3 Were keywords and/or MESH terms stated and where feasible the search strategy provided. |  |  |  |
| 3.4 Was there supplementary searches? |  |  |  |
| 4.1 Were there any restriction for publication type? |  |  |  |
| 4.2 Were there any restriction for language? |  |  |  |
| 5.1 Was a list of included studies provided? |  |  |  |
| 5.2 Was a list of excluded studies provided? |  |  |  |
| 6.1 Was the characteristics of the participants provided? |  |  |  |
| 6.2 Were the characteristics of the interventions provided? |  |  |  |
| 6.3 Was the characteristics of the outcomes provided? |  |  |  |
| 7.1 Was there ‘a priori’ methods of assessment being provided? |  |  |  |
| 7.2 Was a “risk of bias” table show in a graphic form？ |  |  |  |
| 8.1 Were the results of the methodological rigor and scientific quality considered in the analysis of the review? |  |  |  |
| 8.2 Were the results of the methodological rigor and scientific quality considered in the conclusions of the review? |  |  |  |
| 9.1 Was the homogeneity test (i.e. Chi-squared test for homogeneity, I^2^) conducted when pooling results? |  |  |  |
| 9.2Was a random effects model used and/or the clinical appropriateness of combing taken into consideration when heterogeneity exists? |  |  |  |
| 10. Was the likelihood of publication bias assessed? |  |  |  |
| 11.1 Were the sources of support for the systematic review reported？ |  |  |  |
| 11.2 Were the sources of support for the included primary studies reported? |  |  |  |

1. **Statistical analysis**

Extracted data will be presented in the text or as a table to clearly summarize and explain our findings. If any analysis is needed, we will use SPSS to assess the analytical variables.

**References:**

1. Seo HJ, Kim KU: **Quality assessment of systematic reviews or meta-analyses of nursing interventions conducted by Korean reviewers**. *BMC Med Res Methodol* 2012, **12:129.**(doi):10.1186/1471-2288-1112-1129.
2. Momeni A, Lee GK, Talley JR: **The quality of systematic reviews in hand surgery: an analysis using AMSTAR**. *Plastic & Reconstructive Surgery* 2013, **131**(4):831-837.
3. Corbyons K, Han J, Neuberger MM, Dahm P: **Methodological Quality of Systematic Reviews Published in the Urological Literature from 1998 to 2012**. *The Journal of urology* 2015, **194**(5):1374-1379.
4. **Chronic rheumatic conditions. In: World Health Organization.**

<http://www.who.int/chp/topics/rheumatic/en/>. Accessed 15 Jun 2017

1. Tang W, Hu J, Zhang H, Wu P, He H: **Kappa coefficient: a popular measure of rater agreement**. *Shanghai archives of psychiatry* 2015, **27**(1):62-67.
2. Ethgen O, Bruyere O, Richy F, Dardennes C, Reginster JY: **Health-related quality of life in total hip and total knee arthroplasty. A qualitative and systematic review of the literature**. *Journal of Bone & Joint Surgery - American Volume* 2004, **86-A**(5):963-974.
3. Wu XY, Lam VC, Yu YF, Ho RS, Feng Y, Wong CH, Yip BH, Tsoi KK, Wong SY, Chung VC: **Epidemiological characteristics and methodological quality of meta-analyses on diabetes mellitus treatment: a systematic review**. *European journal of endocrinology* 2016, **175**(5):353-360.
4. Sharif MO, Janjua-Sharif FN, Ali H, Ahmed F: **Systematic reviews explained: AMSTAR-how to tell the good from the bad and the ugly.[Erratum appears in Oral Health Dent Manag. 2013 Jun;12(2):119 Note: Sharif, Fyeza N Janjua [corrected to Janjua-Sharif, Fyeza N]]**. *Oral Helath & Dental Management* 2013, **12**(1):9-16.
5. Kung J, Chiappelli F, Cajulis OO, Avezova R, Kossan G, Chew L, Maida CA: **From Systematic Reviews to Clinical Recommendations for Evidence-Based Health Care: Validation of Revised Assessment of Multiple Systematic Reviews (R-AMSTAR) for Grading of Clinical Relevance**. *The open dentistry journal* 2010, **4**:84-91.
6. Pollock A, Farmer SE, Brady MC, Langhorne P, Mead GE, Mehrholz J, van WF: **Interventions for improving upper limb function after stroke**. In: *Cochrane Database of Systematic Reviews.* John Wiley & Sons, Ltd; 2014.

**About the article:**

**Contributions of authors**

WXY wrote the manuscript, WJ designed study and commented on the earlier drafts. LJ contributed towards the conception and design of the study and read and approved the final manuscript.

**Declarations of interest**

There is no conflict of interests.

**Protocol Amendments.**

| **Section of protocol**  Type of amendment | Original version | New version Justification |
| --- | --- | --- |
| Term | mAMSTAR | mod-AMSTAR |
| Study selection and data extraction | 15 characteristics | 10 characteristics, deleted 5 characteristics for they are not statistically independent with the AMSTAR items. |
|  | Total number of participants in SRs | Total number of authors in SRs |
| Statistical analysis | casually described | Add more details in this part |
