# Supplementary material for: Quality assessment of systematic reviews on total hip or knee arthroplasty using mod-AMSTAR
Source: BMC Med Res Methodol. 2018 Mar 16;18:30. doi: 10.1186/s12874-018-0488-8 (PMC5857117; doi:10.1186/s12874-018-0488-8)
Supplement: Supplementary file 2 — Appendix 2. Search strategies: Detailed information on search strategies of this study in Medline, Embase, Cochrane Databases (including HTA, DARE and CDSR), CBM, CNKI, Wang Fang and VIP. (Search strategies). (DOCX 19 kb) [file 12874_2018_488_MOESM2_ESM.docx]

**Electronic Data Bases**

**MEDLINE (Ovid SP)**

1 exp Arthroplasty, Replacement, Knee/ or exp Arthroplasty, Replacement, Hip/

2 (hip$ adj2 (arthroplast$ or implant$ or replace$ or prosthe$ or endoprosthe$)).tw.

3 (knee$ adj2 (arthroplast$ or implant$ or replace$ or prosthe$ or endoprosthe$)).tw.

4 arthroplasty.mp. or exp Arthroplasty, Replacement/ or exp Arthroplasty/

5 Joint Prosthesis.mp. or exp Joint Prosthesis/

6 exp "Prostheses and Implants"/

7 4 or 5 or 6

8 exp Hip/ or exp Hip Joint/ or hip.mp.

9 exp Knee/ or exp Knee Joint/ or knee.mp.

10 8 or 9

11 7 and 10

12 1 or 2 or 3 or 11

13 exp Meta-Analysis as Topic/ or exp Meta-Analysis/

14 Meta-Analysis.pt.

15 (meta-analy$ or metaanaly$ or meta analy$).ab,ti.

16 (systematic$ and (review$ or overview$)).ti,ab.

17 13 or 14 or 15 or 16

18 12 and 17

19 limit 18 to yr="2014 - 2015"

**EMBASE (Ovid SP)**

1 exp Arthroplasty, Replacement, Knee/ or exp Arthroplasty, Replacement, Hip/

2 (hip$ adj2 (arthroplast$ or implant$ or replace$ or prosthe$ or endoprosthe$)).tw.

3 (knee$ adj2 (arthroplast$ or implant$ or replace$ or prosthe$ or endoprosthe$)).tw.

4 arthroplasty.mp. or exp Arthroplasty, Replacement/ or exp Arthroplasty/

5 Joint Prosthesis.mp. or exp Joint Prosthesis/

6 exp "Prostheses and Implants"/

7 4 or 5 or 6

8 exp Hip/ or exp Hip Joint/ or hip.mp.

9 exp Knee/ or exp Knee Joint/ or knee.mp.

10 8 or 9

11 7 and 10

12 1 or 2 or 3 or 11

13 exp Meta-Analysis as Topic/ or exp Meta-Analysis/

14 (meta-analy$ or metaanaly$ or meta analy$).ab,ti.

15 (systematic$ and (review$ or overview$)).ti,ab.

16 13 or 14 or 15

17 12 and 16

18 limit 17 to yr="2014 - 2015"

**Cochrane Database of Systematic Review (CDSR), Database of Abstracts of Reviews of Effects (DARE) and Health Technology Assessment Database (HTA), part of The Cochrane Library:**

#1 Arthroplasty, Replacement, Knee or Arthroplasty, Replacement, Hip

#2 hip and（arthroplast or implant or replace or prosthe or endoprosthe）

#3 knee and（arthroplast or implant or replace or prosthe or endoprosthe）

#4 arthroplasty or Arthroplasty, Replacement or Arthroplasty

#5 Joint Prosthesis or Joint Prosthesis

#6 Prostheses and Implants

#7 #4 or #5 or #6

#8 Hip or Hip Joint

#9 knee or knee joint

#10 #8 or #9

#11 #7 and #10

#12 #1 or #2 or #3 or #11

#13 "Meta-Analysis" or Meta-Analysis

#14 meta-analy or metaanaly or meta analy

#15 systematic and (review or overview)

#16 #13 or #14 or #15

#17 #12 and #16（Publication Year from 2014 to 2015）

**Chinese Domestic Data Bases:**

**Chinese BioMedical Literature Database (CBM):**

1 主题词:关节成形术, 置换, 髋/全部树/全部副主题词

2 主题词:关节成形术, 置换, 膝/全部树/全部副主题词

3 主题词:髋假体/全部树/全部副主题词

4 缺省[智能]:关节置换

5 缺省[智能]:髋关节置换

6 缺省[智能]:膝关节置换

7 缺省[智能]:髋置换

8 缺省[智能]:膝置换

9 缺省[智能]:膝假体

10 缺省[智能]:全髋

11 缺省[智能]:全膝

12 缺省[智能]:人工髋

13 缺省[智能]:人工膝

14 缺省[智能]:关节假体

15 1 or 2 or 3 or 4 or 5 or 6 or 7 or 8 or 9 or 10 or 11 or 12 or 13 or 14

16 主题词:Meta分析/全部树/全部副主题词

17 缺省[智能]:系统评价

18 缺省[智能]:系统综述

19 缺省[智能]:荟萃分析

20 缺省[智能]:meta

21 16 or 17 or 18 or 19 or 20

22 15 and 21 限定 2014-2015

**China National Knowledge Infrastructure (CNKI):**

(SU='髋关节置换术'or SU='膝关节成形术'or SU='髋假体'or KY='关节置换'or KY='髋关节置换'or KY='膝关节置换'or KY='髋置换'or KY='膝置换'or KY='膝假体' or KY='全髋'or KY='全膝'or KY='人工髋'or KY='人工膝'or KY='关节假体')and(SU='Meta分析'or KY='系统评价'or KY='系统综述'or KY='荟萃分析'or KY='meta') and (YE=2014 or YE=2015)

**VIP database:**

(M=髋关节置换术 OR M=膝关节成形术 OR M=髋假体 OR K=关节置换 or K=髋关节置换 OR K=膝关节置换 OR K=髋置换 OR K=膝置换 OR K=膝假体 OR K=全髋 OR K=全膝 OR K=人工髋 OR K=人工膝 OR K=关节假体) AND (M=Meta分析 OR M=系统评价 OR K=系统综述 OR K=荟萃分析 OR K=meta)

限制年份2014-2015

**Wan Fang Data**

(题名或关键词:(关节置换 + 髋关节置换术 + 膝关节置换 + 髋置换 + 膝置换 +膝假体 + 全髋 + 全膝 + 人工髋 + 人工膝 + 关节假体)) * (题名或关键词:(Meta分析 + 系统评价 + 系统综述 + 荟萃分析 + meta)) * Date:2014-2015
