# Supplementary material for: Quality assessment of systematic reviews on total hip or knee arthroplasty using mod-AMSTAR
Source: BMC Med Res Methodol. 2018 Mar 16;18:30. doi: 10.1186/s12874-018-0488-8 (PMC5857117; doi:10.1186/s12874-018-0488-8)
Supplement: Supplementary file 3 — Appendix 3. AMSTAR score and list of included reviews: mod-AMSTAR score for each study and reference information of all included studies. (AMSTAR score and list of included reviews). (DOCX 58 kb) [file 12874_2018_488_MOESM3_ESM.docx]

**Detailed modified AMSTAR scale score for each study**

| **Study** | **1** | **2.1** | **2.2** | **2.3** | **2.4** | **3.1** | **3.2** | **3.3** | **3.4** | **4.1** | **4.2** | | **5.1** | **5.2** | **6.1** | **6.2** | **6.3** | **7.1** | **7.2** | **8.1** | **8.2** | **9.1** | **9.2** | **10** | **11.1** | **11.2** | **SUM** |
| --- | --- | --- | --- | --- | --- | --- | --- | --- | --- | --- | --- | --- | --- | --- | --- | --- | --- | --- | --- | --- | --- | --- | --- | --- | --- | --- | --- |
| **Reviews marched the item in Chinese journal (n=15)** | | | | | | | | | | | | | | | | | | | | | | | | | | | |
| **Min Lingtian**  **2014[1]** | CA | CA | CA | CA | CA | Y | Y | Y | Y | CA | CA | | Y | N | N | N | N | Y | Y | Y | Y | Y | Y | CA | Y | N | 5 |
| **Wang Liguo**  **2014[2]** | CA | Y | Y | CA | CA | Y | Y | Y | Y | CA | CA | | Y | N | Y | Y | N | Y | Y | Y | Y | Y | Y | N | N | N | 5.67 |
| **Xu Jing**  **2015[3]** | CA | Y | Y | CA | CA | Y | Y | Y | N | N | N | | Y | N | Y | Y | N | Y | Y | N | Y | Y | Y | N | N | N | 4.92 |
| **Guo,Jiayong**  **2014[4]** | CA | Y | Y | Y | Y | Y | Y | Y | Y | N | N | | Y | N | Y | Y | Y | Y | Y | Y | N | Y | Y | N | N | N | 6 |
| **Wang Yumin 2015[5]** | CA | Y | Y | CA | CA | Y | Y | Y | N | N | Y | Y | | N | Y | N | N | Y | Y | N | Y | Y | Y | Y | N | N | 6.08 |
| **Zhou Ding**  **2015[6]** | CA | Y | Y | CA | CA | Y | Y | Y | Y | N | CA | | Y | N | Y | Y | Y | Y | Y | N | Y | Y | Y | N | N | N | 5.5 |
| **Liu Weifu**  **2014[7]** | CA | N | N | N | N | Y | Y | Y | Y | N | CA | | Y | N | Y | Y | Y | Y | Y | Y | Y | Y | Y | N | N | N | 5.5 |
| **Liu Binggen**  **2014[8]** | CA | Y | Y | CA | CA | Y | Y | Y | Y | N | N | | Y | N | N | Y | Y | N | Y | N | N | Y | Y | Y | Y | N | 5.67 |
| **Wang Zengliang**  **2014[9]** | CA | Y | Y | Y | N | Y | Y | Y | Y | N | N | | Y | N | Y | Y | Y | N | N | Y | Y | Y | Y | N | Y | N | 5.75 |
| **Zhang Miao**  **2014[10]** | CA | Y | Y | Y | Y | Y | Y | Y | N | N | N | | Y | N | Y | Y | N | Y | N | Y | N | Y | Y | N | N | N | 4.92 |
| **Zhang Huiwei**  **2015[11]** | CA | Y | Y | Y | Y | Y | Y | Y | Y | N | N | | Y | N | Y | Y | Y | Y | Y | N | Y | Y | Y | Y | N | N | 7 |
| **Wang Zhiyong**  **2014[12]** | CA | Y | Y | Y | Y | Y | Y | Y | Y | N | N | | Y | N | Y | Y | Y | Y | Y | Y | Y | Y | Y | Y | N | N | 7.5 |
| **Wu Yue**  **2014[13]** | CA | Y | CA | Y | CA | Y | Y | Y | Y | CA | Y | | Y | N | Y | Y | N | Y | N | N | Y | Y | Y | N | N | N | 5.17 |
| **Zhang Bo**  **2015[14]** | CA | Y | Y | Y | Y | Y | Y | Y | Y | CA | Y | | Y | N | Y | Y | Y | Y | Y | Y | Y | Y | Y | N | Y | N | 7.5 |
| **Han Junliang**  **2015[15]** | CA | Y | Y | CA | CA | Y | Y | Y | N | CA | CA | | Y | N | Y | Y | Y | Y | Y | N | Y | Y | Y | N | Y | N | 5.75 |
| **Reviews marched the item in English journal (n=48)** | | | | | | | | | | | | | | | | | | | | | | | | | | | |
| **Chen, Y.**  **2015[16]** | CA | CA | CA | CA | CA | Y | Y | Y | Y | N | N | | Y | N | Y | Y | Y | Y | Y | Y | N | Y | Y | Y | Y | N | 6.5 |
| **Berstock**  **2014[17]** | Y | Y | Y | Y | Y | Y | Y | Y | Y | Y | Y | | Y | N | Y | Y | Y | Y | Y | Y | Y | Y | Y | Y | Y | N | 10 |
| **Li，Y**  **2014[18]** | CA | Y | Y | Y | Y | Y | Y | Y | N | N | Y | | Y | N | Y | Y | Y | Y | Y | N | Y | Y | Y | N | Y | N | 6.75 |
| **Xu, K**  **2014[19]** | CA | CA | CA | Y | Y | Y | Y | Y | Y | CA | Y | | Y | N | N | Y | N | Y | Y | N | Y | Y | Y | N | Y | N | 5.83 |
| **Liu, Z.**  **2015[20]** | CA | CA | CA | Y | Y | Y | Y | Y | Y | N | N | | Y | N | N | Y | N | N | Y | N | Y | Y | Y | N | Y | N | 4.83 |
| **Zhang, D**  **2015[21]** | CA | Y | Y | Y | CA | Y | Y | Y | CA | N | CA | | Y | N | Y | Y | Y | Y | Y | Y | N | Y | Y | N | N | N | 5.5 |
| **Ni SH**  **2014[22]** | CA | Y | Y | Y | Y | Y | Y | N | Y | CA | Y | | Y | N | N | Y | Y | Y | Y | N | Y | Y | Y | Y | Y | N | 7.42 |
| **Tsertsvadze A 2014[23]** | Y | Y | Y | Y | Y | Y | Y | Y | Y | CA | N | | Y | N | Y | Y | Y | Y | Y | N | Y | Y | Y | N | Y | N | 7.5 |
| **Mihalko, WM**  **2014[24]** | CA | N | N | N | N | Y | Y | Y | Y | N | N | | Y | N | Y | Y | Y | Y | Y | Y | N | N | N | N | Y | N | 4.5 |
| **Shen, C**  **2014[25]** | CA | N | N | Y | Y | Y | Y | Y | Y | N | Y | | Y | N | Y | Y | N | Y | Y | Y | N | Y | Y | Y | Y | N | 7.17 |
| **Yin, S.**  **2015[26]** | CA | Y | Y | Y | Y | Y | Y | Y | Y | N | N | | Y | N | N | Y | Y | Y | Y | Y | N | Y | Y | N | N | N | 5.67 |
| **Wyles, C**  **2015[27]** | CA | Y | CA | N | N | Y | Y | Y | N | N | CA | | Y | N | N | Y | Y | Y | N | CA | CA | Y | Y | N | Y | N | 4.17 |
| **Hu, D.**  **2015[28]** | CA | Y | Y | Y | Y | Y | Y | Y | N | N | N | | Y | N | Y | Y | Y | Y | Y | Y | N | Y | Y | N | Y | Y | 6.75 |
| **Dong, Y.**  **2015[29]** | CA | Y | CA | Y | Y | Y | Y | Y | Y | N | N | | Y | N | Y | Y | N | Y | Y | Y | N | Y | Y | N | N | N | 5.42 |
| **Si, H. B.**  **2015[30]** | CA | Y | Y | Y | Y | Y | Y | Y | Y | N | N | | Y | N | Y | Y | Y | Y | Y | Y | Y | Y | Y | N | Y | N | 7 |
| **Yoon, B.**  **2015[31]** | CA | N | N | Y | CA | Y | Y | Y | CA | N | N | | Y | N | Y | Y | N | Y | Y | Y | N | Y | Y | Y | Y | N | 6.17 |
| **Yue, C**  **2015[32]** | CA | Y | Y | Y | Y | Y | Y | Y | Y | N | CA | | Y | N | Y | Y | Y | Y | Y | N | Y | Y | Y | N | Y | N | 6.5 |
| **Higgins, B. T.**  **2015[33]** | Y | Y | Y | Y | Y | Y | Y | Y | Y | CA | Y | | Y | N | Y | Y | Y | Y | Y | Y | N | Y | Y | N | Y | N | 8 |
| **Berstock, J 2015[34]** | Y | Y | Y | Y | Y | Y | Y | Y | Y | Y | Y | | Y | N | Y | Y | Y | Y | Y | Y | N | Y | Y | Y | Y | N | 9.5 |
| **Cheng**  **2014[35]** | CA | Y | Y | Y | Y | Y | Y | Y | Y | CA | Y | | Y | N | Y | Y | Y | Y | Y | Y | Y | Y | Y | N | Y | N | 7.5 |
| **Li，T**  **2014[36]** | CA | CA | CA | Y | Y | Y | Y | N | Y | Y | Y | | Y | N | Y | Y | Y | Y | Y | Y | Y | Y | Y | N | Y | N | 7.25 |
| **Fan, L.**  **2015[37]** | CA | Y | Y | Y | Y | Y | Y | Y | N | CA | CA | | Y | N | N | Y | Y | Y | Y | Y | N | Y | Y | N | Y | N | 5.92 |
| **Rebal, B.A**  **2014[38]** | Y | CA | CA | Y | Y | Y | N | Y | Y | CA | N | | Y | N | Y | Y | Y | N | N | Y | N | Y | Y | N | Y | N | 5.75 |
| **Study** | **1** | **2.1** | **2.2** | **2.3** | **2.4** | **3.1** | **3.2** | **3.3** | **3.4** | **4.1** | **4.2** | | **5.1** | **5.2** | **6.1** | **6.2** | **6.3** | **7.1** | **7.2** | **8.1** | **8.2** | **9.1** | **9.2** | **10** | **11.1** | **11.2** | **SUM** |
| **Nair R**  **2014[39]** | CA | CA | CA | CA | CA | N | Y | Y | CA | CA | Y | | Y | N | Y | Y | Y | N | N | N | N | N | N | N | Y | N | 3 |
| **Liu, H.W**  **2014[40]** | CA | Y | Y | Y | Y | Y | Y | Y | Y | Y | Y | | Y | N | Y | Y | Y | Y | Y | N | Y | Y | Y | N | Y | N | 7.5 |
| **van der List**  **2015[41]** | CA | Y | Y | CA | CA | Y | Y | Y | CA | N | N | | Y | N | Y | Y | Y | N | N | Y | CA | Y | Y | N | Y | N | 4.75 |
| **Peng, X**  **2015[42]** | CA | Y | CA | Y | Y | Y | Y | Y | Y | N | CA | | Y | N | Y | Y | Y | Y | Y | Y | N | Y | Y | N | N | N | 5.75 |
| **Li, Tao**  **2014[43]** | CA | CA | CA | Y | Y | Y | Y | Y | Y | Y | Y | | Y | N | Y | Y | N | Y | Y | Y | Y | Y | Y | N | N | N | 6.67 |
| **Xu, S**  **2014[44]** | CA | CA | CA | CA | CA | Y | Y | Y | Y | CA | CA | | Y | N | Y | N | N | Y | Y | N | Y | Y | Y | N | Y | N | 4.83 |
| **Li, C**  **2015[45]** | CA | Y | CA | Y | Y | Y | Y | Y | Y | CA | N | | Y | N | Y | Y | N | Y | Y | N | Y | Y | Y | Y | Y | N | 6.92 |
| **Wang, Z**  **2015[46]** | CA | Y | Y | Y | Y | Y | Y | Y | Y | N | N | | Y | N | Y | Y | Y | N | N | N | CA | Y | Y | Y | N | N | 5.5 |
| **Li，N.**  **2015[47]** | CA | Y | CA | Y | CA | Y | Y | Y | Y | N | CA | | Y | N | Y | Y | N | Y | Y | Y | Y | Y | Y | N | Y | N | 6.17 |
| **Li, C**  **2015[48]** | CA | Y | Y | Y | Y | Y | Y | Y | Y | N | CA | | Y | N | Y | Y | N | Y | Y | Y | N | Y | Y | Y | Y | N | 7.17 |
| **Li, C**  **2015[49]** | CA | Y | Y | Y | Y | Y | Y | Y | Y | N | CA | | Y | N | Y | Y | Y | Y | Y | N | Y | Y | Y | Y | Y | N | 7.5 |
| **Jiang, Y.**  **2015[50]** | CA | CA | CA | Y | Y | Y | Y | Y | CA | N | CA | | Y | N | N | Y | N | Y | Y | CA | Y | Y | Y | N | Y | N | 5.08 |
| **Fu, H.**  **2015[51]** | CA | CA | CA | Y | Y | Y | Y | Y | N | N | N | | Y | N | Y | Y | N | Y | Y | Y | N | Y | Y | Y | Y | N | 6.42 |
| **Arirachakara,A.2015[52]** | CA | Y | Y | Y | Y | Y | Y | Y | Y | CA | CA | | Y | N | Y | Y | Y | Y | Y | Y | N | Y | Y | Y | Y | N | 7.5 |
| **Wang, H**  **2014[53]** | CA | CA | CA | Y | Y | Y | CA | Y | Y | CA | Y | | Y | Y | Y | Y | N | Y | Y | N | Y | Y | Y | Y | Y | N | 7.42 |
| **Zhou, Y**  **2015[54]** | CA | CA | CA | CA | CA | Y | Y | Y | Y | N | N | | Y | N | Y | Y | Y | N | N | N | Y | Y | Y | N | Y | N | 4.5 |
| **Verra, W**  **2015[55]** | Y | Y | Y | Y | Y | Y | Y | Y | Y | N | Y | | Y | N | Y | Y | Y | Y | Y | Y | CA | Y | Y | N | Y | N | 8 |
| **Li, N**  **2014[56]** | CA | Y | N | Y | N | Y | Y | Y | Y | N | N | | Y | N | Y | Y | N | Y | Y | Y | N | Y | Y | N | Y | N | 5.67 |
| **Bo, Z. D**  **2014[57]** | CA | CA | CA | Y | Y | Y | Y | Y | Y | Y | Y | | Y | N | Y | Y | N | Y | Y | N | Y | Y | Y | Y | Y | N | 7.67 |
| **Moskal, J.T**  **2014[58]** | CA | Y | Y | CA | CA | Y | Y | Y | Y | N | Y | | Y | Y | Y | Y | N | Y | Y | N | Y | Y | Y | Y | Y | N | 7.67 |
| **Peersman, G**  **2015[59]** | CA | Y | Y | CA | CA | Y | Y | Y | Y | Y | Y | | Y | N | N | N | N | Y | Y | N | Y | Y | Y | N | Y | N | 6 |
| **Li, Y.L**  **2014[60]** | CA | Y | Y | Y | Y | Y | Y | Y | Y | N | Y | | Y | N | Y | Y | Y | Y | Y | Y | Y | Y | Y | N | Y | N | 7.5 |
| **Xie, X**  **2014[61]** | CA | Y | Y | Y | Y | Y | Y | Y | Y | CA | N | | Y | Y | N | Y | Y | Y | Y | N | Y | Y | Y | N | Y | N | 6.67 |
| **Cheng, T**  **2015[62]** | CA | Y | Y | Y | CA | Y | Y | Y | Y | Y | Y | | Y | Y | Y | Y | Y | Y | Y | N | Y | Y | Y | N | Y | N | 7.75 |
| **Nieuwenhuijse MJ,2014[63]** | CA | Y | Y | Y | Y | Y | Y | Y | Y | CA | N | | Y | N | Y | Y | Y | Y | Y | Y | N | Y | Y | Y | Y | N | 7.5 |

**Y—Yes; N—No; CA—Can’t Answer**

**Included studies**

1. Min Lingtian WW, Wang Weijun, Wu Mingda,Yuan Tao: **Clinical efficacy of minimally invasive exposure for total hip arthroplasty: a systematic review and Meta-analysis**. *Chin J Bone Joint Injury* 2014(02).

2. Wang Liguo Lx, Li Leiming,Meng Fanhe,Wang Jun,Bian Yan,Du Zhangzhen,Fan guangyu: **Meta Analysis ofthe Effect of Soft Tissue Repair on Prevention ofDislocation after Primary Total Hip Arthroplasty**. *journal of China Medical University* 2014(09).

3. Xu Jing XH, Zhao Jianning, Zhang Lei.: **A meta-analysis of application results of hydroxyapatite-coated femoral implants in total hip arthroplasty**. *Chinese Journal of Bone and Joint* 2015.

4. Guo J: **Hydroxyapatite-coated hip prosthesis in primary total hip arthroplasty, a meta-analysis** *.* Anhui Medical University 2014.

5. Yumin W: **Comparison of cemented versus uncemented fixation in total hip replacement: a meta-analysis of available evidence**. *Orthopedic Journal of China* 2015(24).

6. Zhou Ding ZMHYZQ: **Meta- analysis of cemented versus uncemented fixation of femoral components in primary total hip arthroplasty**. *Chinese J Joint Surg(Electronic Edition）* 2014.

7. LiuWeifu: **Patellar Resurfacing in Total Knee Arthroplasty -A Systematic Review and Meta-analysis**. Fujian Medical University Master's Thesis; 2014.

8. Binggen L, Qingjiang P: **Meta-analysis of therapeutic effects of computer-assisted navigation versus conventional total knee arthroplasty**. *Chinese Journal of Tissue Engineering Research* 2014(40):6542-7.

9. Wang Zengliang ZL, Zhao Jiaguo: **Meta-analysis of limb and prosthesis alignment restoration after navigated total knee arthroplasty versus conventional total knee arthroplasty**. *Chinese Journal of Tissue Engineering Research* 2014(35):5707-14.

10. Liling ZMBBCYZSL: **Comparison of minimal- incision midvastus and standard medial parapatellar approaches for total**

**knee arthroplasty: meta-analysis**. *Chinese J Joint Surg(Electronic Edition）* 2014.

11. Zhang Huiwei LK, Fan Yaling,Wang Yongcai,Yan Bing,Wang Hongchuan,Jiang Junwei: **Comparison of Knee Flexion Degree after High-flexion versus Standard Total Knee Arthroplasty: A Meta-analysis**. *West China Medical Journal* 2015.

12. Zhiyong W: **Comparison of Cemented and Cementless Fixation in Total Knee Arthroplasty of A Meta-Analysis**. Shanxi Medical University; 2014.

13. WU Yue YB, Liu Haibing,Li Yan,Zhang Zhongqiang,Tang Liang: **Comparison of Fixed-Bearing and Mobile-Bearing in Bilateral Total Knee Replacement: a Systematic Review**. *Medical Science Journal of Central South China* 2014.

14. Zhang Bo DW, Guo Li,Tian ruiyuan, Li Pengcui,Wei xiaocun: **Fixed -bearing knee prostheses or mobile-bearing knee prostheses: a meta-analysis on clinical outcome of total knee arthroplasty**. *Orthopedic Journal of China* 2015, **23**(19):1774-8.

15. Wei HJDWGYGLLP, Xiaochun: **Meta-analysis on clinical outcomes comparison of mobile- bearing and fixed- bearing total knee arthroplasties for knee arthritis**. *Chinese J Joint Surg(Electronic Edition）* 2015(3):370-6.

16. Chen YL, Lin T, Liu A, Shi MM, Hu B, Shi ZL, Yan SG: **Does hydroxyapatite coating have no advantage over porous coating in primary total hip arthroplasty? A meta-analysis**. *Journal of Orthopaedic Surgery* 2015, **10**:21.

17. Berstock JR, Blom AW, Beswick AD: **A systematic review and meta-analysis of the standard versus mini-incision posterior approach to total hip arthroplasty**. *Journal of Arthroplasty* 2014, **29**(10):1970-82.

18. Li YL, Jia J, Wu Q, Ning GZ, Wu QL, Feng SQ: **Evidence-based computer-navigated total hip arthroplasty: an updated analysis of randomized controlled trials**. *European journal of orthopaedic surgery & traumatologie* 2014, **24**(4):531-8.

19. Xu K, Li Y, Zhang H, Wang C, Xu Y, Li Z: **Computer navigation in total hip arthroplasty: a meta-analysis of randomized controlled trials**. *International journal of surgery* 2014, **12**(5):528-33.

20. Liu Z, Gao Y, Cai L: **Imageless navigation versus traditional method in total hip arthroplasty: A meta-analysis**. *International Journal Of Surgery* 2015, **21**:122-7.

21. Zhang D, Chen L, Peng K, Xing F, Wang H, Xiang Z: **Effectiveness and safety of the posterior approach with soft tissue repair for primary total hip arthroplasty: a meta-analysis**. *Orthopaedics & traumatology, surgery & research* 2015, **101**(1):39-44.

22. Ni SH, Guo L, Jiang TL, Zhao J, Zhao YG: **Press-fit cementless acetabular fixation with and without screws**. *International Orthopaedics* 2014, **38**(1):7-12.

23. Tsertsvadze A, Grove A, Freeman K, Court R, Johnson S, Connock M, Clarke A, Sutcliffe P: **Total hip replacement for the treatment of end stage arthritis of the hip: a systematic review and meta-analysis**. *PLoS ONE [Electronic Resource]* 2014, **9**(7):e99804.

24. Mihalko WM, Wimmer MA, Pacione CA, Laurent MP, Murphy RF, Rider C: **How have alternative bearings and modularity affected revision rates in total hip arthroplasty?** *Clinical Orthopaedics & Related Research* 2014, **472**(12):3747-58.

25. Shen C, Tang ZH, Hu JZ, Zou GY, Xiao RC, Yan DX: **Does cross-linked polyethylene decrease the revision rate of total hip arthroplasty compared with conventional polyethylene? A meta-analysis**. *Orthopaedics & traumatology, surgery & research* 2014, **100**(7):745-50.

26. Yin S, Zhang D, Du H, Yin Z, Qiu Y: **Is there any difference in survivorship of total hip arthroplasty with different bearing surfaces? A systematic review and network meta-analysis**. *International Journal of Clinical and Experimental Medicine* 2015, **8**(11):21871-85.

27. Wyles CC, Jimenez-Almonte JH, Murad MH, Norambuena-Morales GA, Cabanela ME, Sierra RJ, Trousdale RT: **There Are No Differences in Short- to Mid-term Survivorship Among Total Hip-bearing Surface Options: A Network Meta-analysis**. *Clinical Orthopaedics & Related Research* 2015, **473**(6):2031-41.

28. Hu D, Yang X, Tan Y, Alaidaros M, Chen L: **Ceramic-on-ceramic versus ceramic-on-polyethylene bearing surfaces in total hip arthroplasty**. *Orthopedics* 2015, **38**(4):e331-e8.

29. Dong YL, Li T, Xiao K, Bian YY, Weng XS: **Ceramic on Ceramic or Ceramic-on-polyethylene for Total Hip Arthroplasty: A Systemic Review and Meta-analysis of Prospective Randomized Studies**. *Chinese Medical Journal* 2015, **128**(9):1223-31.

30. Si HB, Zeng Y, Cao F, Pei FX, Shen B: **Is a ceramic-on-ceramic bearing really superior to ceramic-on-polyethylene for primary total hip arthroplasty? A systematic review and meta-analysis of randomised controlled trials**. *Hip International* 2015, **25**(3):191-8.

31. Yoon BH, Ha YC, Lee YK, Koo KH: **Postoperative Deep Infection After Cemented Versus Cementless Total Hip Arthroplasty: A Meta-Analysis**. *Journal of Arthroplasty* 2015, **30**(10):1823-7.

32. Yue C, Kang P, Pei F: **Comparison of Direct Anterior and Lateral Approaches in Total Hip Arthroplasty: A Systematic Review and Meta-Analysis (PRISMA)**. *Medicine* 2015, **94**(50):e2126.

33. Higgins BT, Barlow DR, Heagerty NE, Lin TJ: **Anterior vs. posterior approach for total hip arthroplasty, a systematic review and meta-analysis**. *Journal of Arthroplasty* 2015, **30**(3):419-34.

34. Berstock JR, Blom AW, Beswick AD: **A systematic review and meta-analysis of complications following the posterior and lateral surgical approaches to total hip arthroplasty**. *Annals of the Royal College of Surgeons of England* 2015, **97**(1):11-6.

35. Cheng T, Zhu C, Guo Y, Shi S, Chen D, Zhang X: **Patellar denervation with electrocautery in total knee arthroplasty without patellar resurfacing: a meta-analysis**. *Knee surgery, sports traumatology, arthroscopy : official journal of the ESSKA* 2014, **22**(11):2648-54.

36. Li T, Zhou L, Zhuang Q, Weng X, Bian Y: **Patellar denervation in total knee arthroplasty without patellar resurfacing and postoperative anterior knee pain: a meta-analysis of randomized controlled trials**. *Journal of Arthroplasty* 2014, **29**(12):2309-13.

37. Fan L, Ge Z, Zhang C, Li J, Yu Z, Dang X, Wang K: **Circumferential electrocautery of the patella in primary total knee replacement without patellar replacement: a meta-analysis and systematic review**. *Scientific Reports* 2015, **5**:9393.

38. Rebal BA, Babatunde OM, Lee JH, Geller JA, Patrick DA, Jr., Macaulay W: **Imageless computer navigation in total knee arthroplasty provides superior short term functional outcomes: a meta-analysis**. *Journal of Arthroplasty* 2014, **29**(5):938-44.

39. Nair R, Tripathy G, Deysine GR: **Computer navigation systems in unicompartmental knee arthroplasty: a systematic review**. *American Journal of Orthopedics (Chatham, Nj)* 2014, **43**(6):256-61.

40. Liu HW, Gu WD, Xu NW, Sun JY: **Surgical approaches in total knee arthroplasty: a meta-analysis comparing the midvastus and subvastus to the medial peripatellar approach**. *Journal of Arthroplasty* 2014, **29**(12):2298-304.

41. van der List JP, McDonald LS, Pearle AD: **Systematic review of medial versus lateral survivorship in unicompartmental knee arthroplasty**. *knee* 2015, **22**(6):454-60.

42. Peng X, Zhang X, Cheng T, Cheng M, Wang J: **Comparison of the quadriceps-sparing and subvastus approaches versus the standard parapatellar approach in total knee arthroplasty: A meta-analysis of randomized controlled trials Orthopedics and biomechanics**. *BMC Musculoskeletal Disorders* 2015, **16**(1).

43. Li, Tao;Zhuang, Qianyu;Xiao, Ke;Zhou, Lei;Weng, Xisheng: **Comparison of the clinical and radiological outcomes following midvastus and medial parapatellar approaches for total knee arthroplasty: a meta-analysis**. *Chinese Medical Journal* 2014.

44. Xu SZ, Lin XJ, Tong X, Wang XW: **Minimally invasive midvastus versus standard parapatellar approach in total knee arthroplasty: a meta-analysis of randomized controlled trials**. *PLoS ONE [Electronic Resource]* 2014, **9**(5):e95311.

45. Li C, Zeng Y, Shen B, Kang P, Yang J, Zhou Z, Pei F: **A meta-analysis of minimally invasive and conventional medial parapatella approaches for primary total knee arthroplasty**. *Knee Surgery, Sports Traumatology, Arthroscopy* 2015, **23**(7):1971-85.

46. Wang Z, Wei M, Zhang Q, Zhang Z, Cui Y: **Comparison of High-Flexion and Conventional Implants in Total Knee Arthroplasty: A Meta-Analysis**. *Medical Science Monitor* 2015, **21**:1679-86.

47. Li N, Li J, Li P, Wang D, Liu M, Xia L: **Standard versus high-flexion posterior stabilized total knee prostheses**. *Orthopedics* 2015, **38**(3):e206-12.

48. Li C, Shen B, Yang J, Zhou Z, Kang P, Pei F: **Do patients really gain outcome benefits when using the high-flex knee prostheses in total knee arthroplasty? A meta-analysis of randomized controlled trials**. *Journal of Arthroplasty* 2015, **30**(4):580-6.

49. Li C, Zeng Y, Shen B, Yang J, Zhou Z, Kang P, Pei F: **Patients achieved greater range of movement when using high-flexion implants**. *Knee Surgery, Sports Traumatology, Arthroscopy* 2015, **23**(6):1598-609.

50. Jiang Y, Yao JF, Xiong YM, Ma JB, Kang H, Xu P: **No Superiority of High-Flexion vs Standard Total Knee Arthroplasty: An Update Meta-Analysis of Randomized Controlled Trials**. *Journal of Arthroplasty* 2015, **30**(6):980-6.

51. Fu H, Wang J, Zhang W, Cheng T, Zhang X: **No clinical benefit of high-flex total knee arthroplasty. A meta-analysis of randomized controlled trials**. *Journal of Arthroplasty* 2015, **30**(4):573-9.

52. Arirachakaran A, Wande T, Pituckhanotai K, Predeeprompan P, Kongtharvonskul J: **Clinical outcomes after high-flex versus conventional total knee arthroplasty**. *Knee Surgery, Sports Traumatology, Arthroscopy* 2015, **23**(6):1610-21.

53. Wang H, Lou H, Zhang H, Jiang J, Liu K: **Similar survival between uncemented and cemented fixation prostheses in total knee arthroplasty: a meta-analysis and systematic comparative analysis using registers**. *Knee Surgery, Sports Traumatology, Arthroscopy* 2014, **22**(12):3191-7.

54. Zhou Y, Li L, Zhou Q, Yuan S, Wu Y, Zhao H, Wu H: **Lack of efficacy of prophylactic application of antibiotic-loaded bone cement for prevention of infection in primary total knee arthroplasty: results of a meta-analysis**. *Surgical Infections* 2015, **16**(2):183-7.

55. Verra WC, Van Den Boom LGH, Jacobs WCH, Schoones JW, Wymenga AB, Nelissen RGHH: **Similar outcome after retention or sacrifice of the posterior cruciate ligament in total knee arthroplasty: A systematic review and meta-analysis**. *Acta Orthopaedica* 2015, **86**(2):195-201.

56. Li N, Tan Y, Deng Y, Chen L: **Posterior cruciate-retaining versus posterior stabilized total knee arthroplasty: a meta-analysis of randomized controlled trials**. *Knee Surgery, Sports Traumatology, Arthroscopy* 2014, **22**(3):556-64.

57. Bo ZD, Liao L, Zhao JM, Wei QJ, Ding XF, Yang B: **Mobile bearing or fixed bearing? A meta-analysis of outcomes comparing mobile bearing and fixed bearing bilateral total knee replacements**. *Knee* 2014, **21**(2):374-81.

58. Moskal JT, Capps SG: **Rotating-platform TKA no different from fixed-bearing TKA regarding survivorship or performance: a meta-analysis**. *Clinical Orthopaedics & Related Research* 2014, **472**(7):2185-93.

59. Peersman G, Stuyts B, Vandenlangenbergh T, Cartier P, Fennema P: **Fixed- versus mobile-bearing UKA: a systematic review and meta-analysis**. *Knee Surgery, Sports Traumatology, Arthroscopy* 2015, **23**(11):3296-305.

60. Li YL, Wu Q, Ning GZ, Feng SQ, Wu QL, Li Y, Hao Y: **No difference in clinical outcome between fixed- and mobile-bearing TKA: a meta-analysis**. *Knee Surgery, Sports Traumatology, Arthroscopy* 2014, **22**(3):565-75.

61. Xie X, Lin L, Zhu B, Lu Y, Lin Z, Li Q: **Will gender-specific total knee arthroplasty be a better choice for women? A systematic review and meta-analysis**. *European journal of orthopaedic surgery & traumatologie* 2014, **24**(8):1341-9.

62. Cheng T: **No clinical benefit of gender-specific total knee arthroplasty: A systematic review and meta-analysis of 6 randomized controlled trials. Author reply**. *Acta Orthopaedica* 2015, **86**(2):274-5.

63. Nieuwenhuijse MJ, Nelissen RG, Schoones JW, Sedrakyan A: **Appraisal of evidence base for introduction of new implants in hip and knee replacement: a systematic review of five widely used device technologies**. *BMJ* 2014, **349**:g5133.
